# *XAS3DLive*: an interactive X-ray absorption spectroscopy simulation/fitting platform for atomic 3D structure characterization

**DOI:** 10.1107/S1600577526008969

**Published:** 2026-09-15

**Authors:** Xueqi Song, Haodong Yao, Haifeng Zhao

**Affiliations:** a Multi-disciplinary Research Division Institute of High Energy Physics 19B Yuquan Road Shijingshan District Beijing100049 People’s Republic of China; b Institute of High Energy Physics University of Chinese Academy of Sciences 19A Yuquan Road Shijingshan District Beijing100049 People’s Republic of China; ESRF – The European Synchrotron, France

**Keywords:** X-ray absorption spectroscopy analysis platform, 3D structure characterization, machine learning, fitting, 3D structure visualization

## Abstract

We present *XAS3DLive*, an integrated platform for X-ray absorption spectroscopy (XAS) analysis that unifies machine-learning-driven XAS simulation, 3D structure visualization and editing, and experimental XAS fitting into a single interactive environment. The platform enables 3D structure visualization and manipulation, spectral analysis, and structure fitting to be performed by a web browser. Specifically, the machine-learning-based model enables rapid XAS simulation of spectral changes in response to structural variations; the 3D structure visualization and editing supports intuitive, atomic-level manipulation and precise adjustments; the global optimization algorithm helps to achieve automatically a detailed 3D structure of a material in an experimental XAS fitting. Multiple toolkits are provided to facilitate XAS data analysis, such as multi-spectrum comparison, energy shift, difference spectrum analysis, and quantitative evaluation. With the machine-learning model trained for inorganic complex systems with 3*d* transition metals, *XAS3DLive* is now available for *K*-edge XANES simulation of all ten 3*d* transition metals, providing a focused capability for a wide range of commonly studied transition-metal-based materials.*XAS3DLive* has been used for the structure analysis of Mn-doped Co_3_O_4_ and the Jahn–Teller effect has been successfully confirmed in experimental XAS fitting. This platform significantly lowers the barrier to entry for XAS analysis, improves the efficiency of XAS simulation and experimental XAS fitting, and enhances an understanding of the structure–XAS relationship through intuitive interaction.

## Abstract 2

An interactive X-ray absorption spectroscopy (XAS) simulation/fitting platform for atomic 3D structure characterization integrates machine-learning-driven XAS simulation, 3D structure visualization and editing, and experimental XAS fitting into one unity for fast and user-friendly analysis.

## 1. Introduction

X-ray absorption spectroscopy (XAS) is one of most popular experimental tools used at synchrotron radiation facilities, providing element-specific insights into electronic structure and local atomic environments (Rehr & Albers, 2000 ▸). XAS is generally divided into two spectral regions—X-ray absorption near-edge structure (XANES) and extended X-ray absorption fine structure (EXAFS) (Joly *et al.*, 2009 ▸). The former, which includes more fine structure than the latter, can be analyzed carefully to derive the valence structure of an atom of interest and the three-dimensional atomic geometry of a system, which is essential to understand the macroscopic behavior of advantageous materials. Several software packages based on different frameworks, such as real-space Green function multiple scattering theory (*FEFF*, for example; Rehr *et al.*, 2010 ▸) and density functional theory (DFT)/time-dependent density functional theory (TDDFT) (*FDMNES*, *ORCA*, *etc.*) (Joly, 2001 ▸; Bunău & Joly, 2009 ▸; Neese, 2012 ▸), have been developed and optimized continuously in recent decades; the former works well for extended solids due to its efficient multiple-scattering formalism (Rehr *et al.*, 2010 ▸), where a detailed *n*-leg scattering path presentation offers physical insight into why peaks in a spectrum exist, while TDDFT-based approaches are more suitable for finite molecular and coordination-complex systems, where localized electronic excitations are of primary interest. Generally, the simulation of XANES with these software packages demands careful 3D modeling of a system and reasonable setting of non-structure parameters, and then arrives at the convergence of simulated and experimental data (Zhan *et al.*, 2017 ▸). An optimization algorithm is usually used outside of the framework of XANES simulation to find the optimal structure for a material by comparing the experimental data—we call it fitting; one of the most popular used packages is *MXAN* (Benfatto *et al.*, 2001 ▸), which is very suitable for the extraction of local structure around a metal atom in biological systems. The fitting is usually quite time-consuming—the simulation of XANES for a given geometry of a system with about 100 atoms generally takes three minutes or more; a fitting for searching several thousand possible structures is reasonable. Though parallel algorithms and high-performance computing improve the fitting efficiency greatly, achieving final results on the minute scale is still a work in progress. Moreover, XANES fitting is generally a black box—it is difficult for users to follow the modification of a geometric structure of a material to check the corresponding behavior of the spectrum, then to understand the relationship between the fine structures in the spectral line and the external physical performances of the material.

Since Timoshenko *et al.* (2017 ▸) first reported on the use of XANES and neural networks (NNs) for refining the 3D geometry of metal catalysts, supervised machine-learning (Hastie *et al.*, 2009 ▸) approaches have been increasingly employed to uncover the underlying relationship between the XANES features and the material geometry. With the advantage of machine learning (ML) (Udousoro, 2020 ▸) in fast simulation, it is possible to derive the symmetry of a cluster, the bond length around an absorber (Torrisi *et al.*, 2020 ▸), the coordinate number (Zheng *et al.*, 2020 ▸), and even the radial distribution function (RDF) (Timoshenko *et al.*, 2018 ▸) quickly from ‘spectrum to structure’ ML models (with XAS as input and structure as output) after sufficient training on well organized datasets. Most existing approaches, however, yield zero-dimensional (values) or one-dimensional (lines, RDF for example) output (Han *et al.*, 2025 ▸), while the extraction of a richer local geometry around an absorber—such as relative Cartesian coordinates—remains a significant challenge for such ‘spectroscopy to structure’ ML models. Recently, graph neural networks (GNNs) (Wu *et al.*, 2021 ▸) have shown their advantage in accurate XANES simulation for solids and molecules, taking the atoms as nodes and chemical bonds or interactions as edges, which is very suitable for the modeling of a material’s structure. With this ‘structure to spectrum’ ML model, it is possible to obtain the 3D structure of a material by adding an optimization algorithm loop outside for fitting. Moreover, by reorganizing the architecture of GNNs and optimizing the feature function in the message passing scheme, as we did in the XAS3D ML model (Zhan *et al.*, 2025 ▸; Zhan & Geng, 2026 ▸), it is possible to generalize the model to predict the XANES for element-dependent systems for all structures. We have shown its accuracy in *K*-edge XANES simulations and its robustness in iron-contained systems, and application of the XAS3D model to Mn-doped Co_3_O_4_ has shown its ability to observe the Jahn–Teller effect in this complex system—the fitting time is reduced from several days in conventional treatment based on multiple scattering theory to several minutes.

Compared with conventional first-principles methods such as *FEFF* and *ORCA*, GNN-based models do not explicitly describe XAS spectra in terms of individual multiple-scattering or electronic excitation processes and therefore cannot provide the same level of physical interpretability. Nevertheless, their major advantage is computational efficiency. Once trained, a GNN model can simulate the XAS of a given structure within the sub-second, several orders of magnitude faster than conventional first-principles calculations. With this in mind, the ML model can achieve the simulation of XANES for a given geometry within the sub-second, making it possible to speed up XANES fitting by replacing the XANES simulation step with a well trained ML model. Moreover, it helps to open the fitting black box by extracting every step at the movements of the atoms in a system, then displaying them through 3D visualization, accompanied by the corresponding spectra, just like watching a movie. With the advantage of the XANES ML model, selection and dragging of an atom in a system and finding out how it affects the peaks or fine structures in a spectrum is no longer a dream—it is definitely helpful in improving the users’ understanding of the intrinsic relationship between the atomic 3D structure of a system and the detected XANES line. All these works have been completed in our XANES simulation/fitting platform, *XAS3DLive* (https://xas3dlive.ihep.ac.cn)—a comprehensive presentation of which is given in this manuscript.

Recently, several software packages and web-based platforms have been developed to facilitate XAS simulations. *Lightshow* provides a convenient way to prepare input files for specified software [*FEFF*, *XSpectra* (Taillefumier *et al.*, 2002 ▸), *OCEAN* (Vinson *et al.*, 2011 ▸), *exciting* (Vorwerk *et al.*, 2019 ▸) and *VASP* (Karsai *et al.*, 2018 ▸)], thereby simplifying the transition of 3D structure in input files for different software (Carbone *et al.*, 2023 ▸). *Web-CONEXS* supports a web-based automated workflow that integrates multiple XAS simulation programs, including *ORCA*, *FDMNES* and *Quantum ESPRESSO* (Giannozzi *et al.*, 2009 ▸), with online high performance computing resources, enabling users to effortlessly execute XAS simulation via pre-set parameter templates and an accessible browser environment (Elliott *et al.*, 2024 ▸). Both *Lightshow* and *Web-CONEXS* make the simulation significantly more convenient for users, but contribute little to the speed of calculation. *LightshowAI* goes further—it provides a web-based environment for rapid XAS simulation based on the deep-learning model *OmniXAS* (Kharel *et al.*, 2025 ▸). With the advantage of an ML model in simulation speed, *LightshowAI* can immediately output the XAS line when a user inputs the structure of a material. The structure is shown as a three-dimensional view but cannot be edited, so it is difficult for users to find the intrinsic relationship between the material geometric structure and the fine structure in the spectrum in this static calculation. Clearly, there is still considerable room for optimization in terms of user experience, particularly in enabling more dynamic and interactive structural manipulation.

This manuscript is organized as follows. The architecture of the XANES ML model and its performance in XANES simulation are given in the *Methodology and implementation* section[Sec sec2]; technical details of a panel for 3D structure visualization and editing of a system are also introduced therein. The **XAS3DLive* platform and its functionalities* section[Sec sec3] gives all details of the *XAS3DLive* platform, including toolkits for energy alignment and structure transfer for input materials. The experimental XANES for the Mn-doped Co_3_O_4_ system is fitted in the *Application* section[Sec sec4] to show the performance of the *XAS3DLive* platform. The *Discussion* section[Sec sec5] follows the *Application* section, and considers the limitations and applicability of the proposed method. Finally comes the *Conclusion*[Sec sec6].

## 2. Methodology and implementation

### 2.1. Machine-learning-based model for rapid XANES simulation

*XAS3DLive* employs an ML-driven approach for rapid XANES simulation, built upon our previously developed XAS3D model. XAS3D is a customized 3D GNNs model specified for detection of 3D local structure coordinates for solid materials and nanoclusters with any configurations; it helps improve the accuracy of outputted XANES.

The XAS3D model is composed of one embedding layer, several interaction layers and one pooling layer to output XANES data. The embedding layer encodes the atomic properties into an initial node attribute vector, which is then updated iteratively by a graph convolution in the following interaction layers. The key geometric features, such as the interatomic distances, bond angles, and dihedral angles, are first extracted from the atomic coordinates of the system and then subsequently encoded via learnable feature functions. These features are combined with those of neighboring atoms through a message-passing scheme to update atomic representations, enabling the progressive capture of complex nonlinear relationships between local atomic environments and spectroscopic responses. After successive updates across the interaction layers, the atomic representations are aggregated via sum pooling to form a global structural descriptor, which is finally mapped to the simulated XANES. The XAS3D model is trained on a paired ‘structure-XANES’ dataset, where the 3D structures of a system containing 3*d* transition metals from Sc to Zn are taken from the Cambridge Structural Database (Groom *et al.*, 2016 ▸) and Materials Project repository (Jain *et al.*, 2013 ▸). Latin hypercube sampling is used to extend datasets whose structures are limited. The simulated XANES were generated using *FDMNES* where the calculations were performed in the multiple-scattering mode using real Hedin–Lundqvist parametrization within the local density approximation (LDA) to describe the exchange–correlation potential. A cluster with a radius of 5 Å centered on the absorbing atom was adopted, and was found to be sufficient to ensure convergence of the self-consistent cycle involving the charge density, Dyson equation, and Green’s functions. This formalism makes it particularly suitable for the simulation of *K*-edge spectra for all elements, as well as *L*_2,3_-edge spectra for heavier elements. Based on this physically grounded approach, the generated dataset provides reliable structure–spectrum pairs for model training. The resulting XAS3D model demonstrates high accuracy and stability in XANES simulations, as reported in previous work (Zhan *et al.*, 2025 ▸) and in a recent study (Zhan & Geng, 2026 ▸). While both models employ the same network architecture, the 2025 version was developed as an element-specific model, whereas the 2026 version is a generalized model trained to simulate *K*-edge XANES across multiple 3*d* transition metals. The implementation of the universal XAS3D model, including the source code, model weights, and training scripts, has been made publicly available at https://github.com/zhanfeichem/XAS3Duniversal, enabling reproducibility and further development by the community.

### 2.2. Visualization and editing of atomic 3D structure

**Figure 1:**
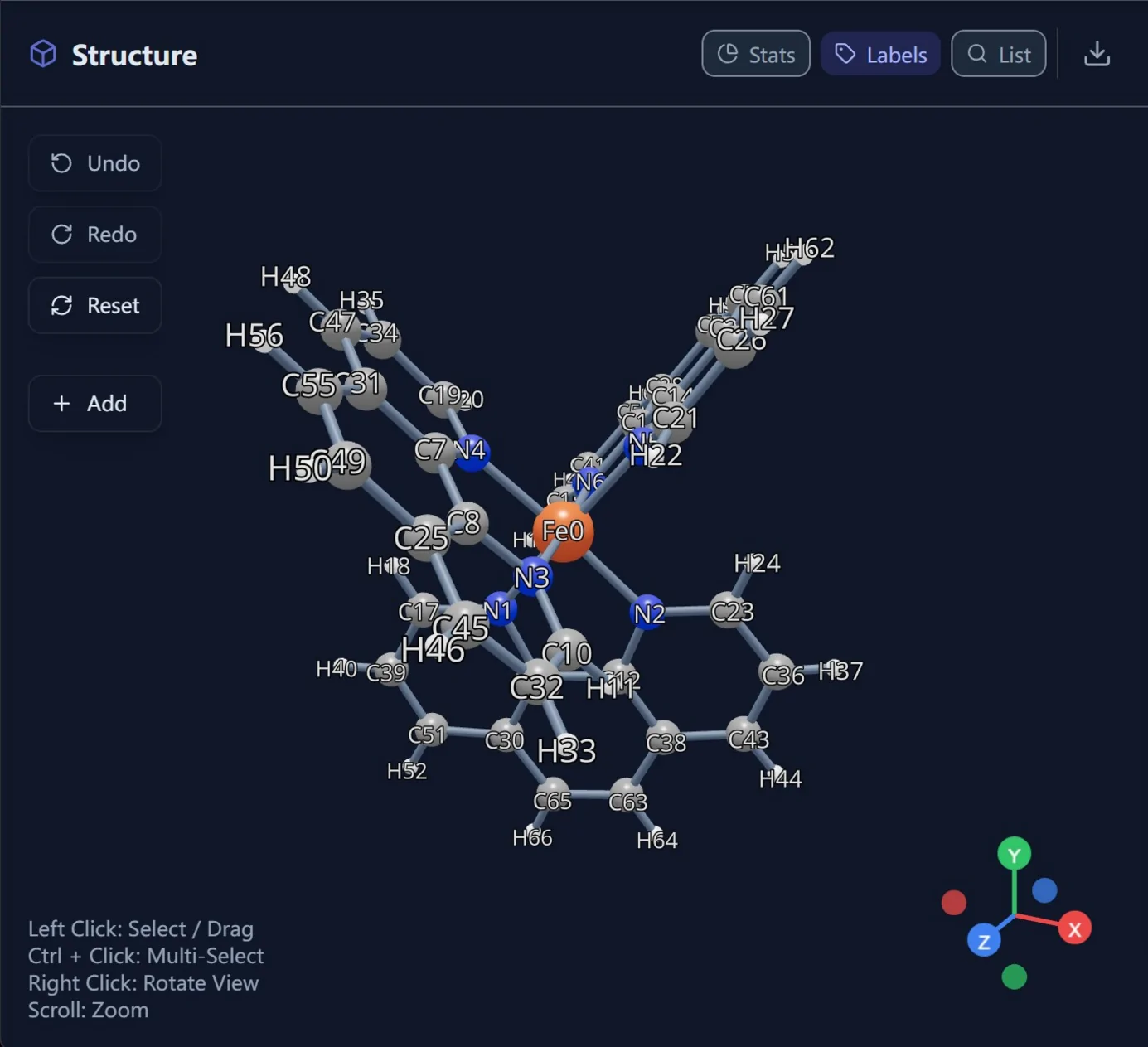
Structure panel diagram: the 3D structure is visualized with a ball-and-stick model, colored according to the CPK color scheme, and includes atomic labels and axis indicators.

*XAS3DLive* establishes a 3D Structure panel (as shown in Fig. 1 ▸), which enables visualization and editing of atomic structures. The panel is built on the Three.js library, which supports efficient rendering and real-time interaction within a web browser and allows for smooth manipulation of complex structures. This design provides a foundation for structure editing and integrated spectral analysis, while also enabling interfacing with other modules.

Three.js, an open-source JavaScript library for browser-based 3D graphics (created by Ricardo Cabello), was employed to visualize and manipulate atomic structures in *XAS3DLive*. It provides interactive operations such as rotation, translation, and scaling, together with flexible rendering of atoms and chemical bonds. Combined with the platform’s real-time spectral simulation engine, Three.js enables synchronized 3D structure editing and immediate XANES updates, providing an intuitive interactive environment for XAS analysis.

### 2.3. Optimization algorithm used in experimental XANES fitting algorithm

*XAS3DLive* adopts a deterministic global optimization algorithm called Dividing Rectangles (DIRECT) (Jones *et al.*, 1993 ▸), which helps to find the best structure of a system for which the simulated XANES reaches closest to the experimental data. This algorithm works by adaptively splitting the parameter space, repeatedly dividing the search region into smaller hyper-rectangles. In this way, it keeps a balance between global exploration and local refinement, making it easier to approach the global optimum. As a widely adopted optimization algorithm, DIRECT can significantly reduce the number of function evaluations while still maintaining stable convergence, which improves both the efficiency and the reliability of the XAS fitting process.

## 3. *XAS3DLive* platform and its functionalities

### 3.1. Platform overview

We developed an online XAS simulation/fitting platform *XAS3DLive*. With the integration of a GNN-based ML model for XANES simulation, an interactive interface for atomic 3D structure visualization/modification, and an optimization algorithm for fitting, *XAS3DLive* supports fast XANES simulations when the geometry of a system is defined, the modification of the material structure in a 3D view and showing up of the XANES line immediately. This helps users to explore the evolution of how the geometric structure around an absorber affects the fine structure within the spectral line. The ML-based XAS simulator combining the optimization algorithm provides a fast 3D structural characterization when the experimental data are provided. *XAS3DLive* is now available online (https://xas3dlive.ihep.ac.cn).

**Figure 2:**
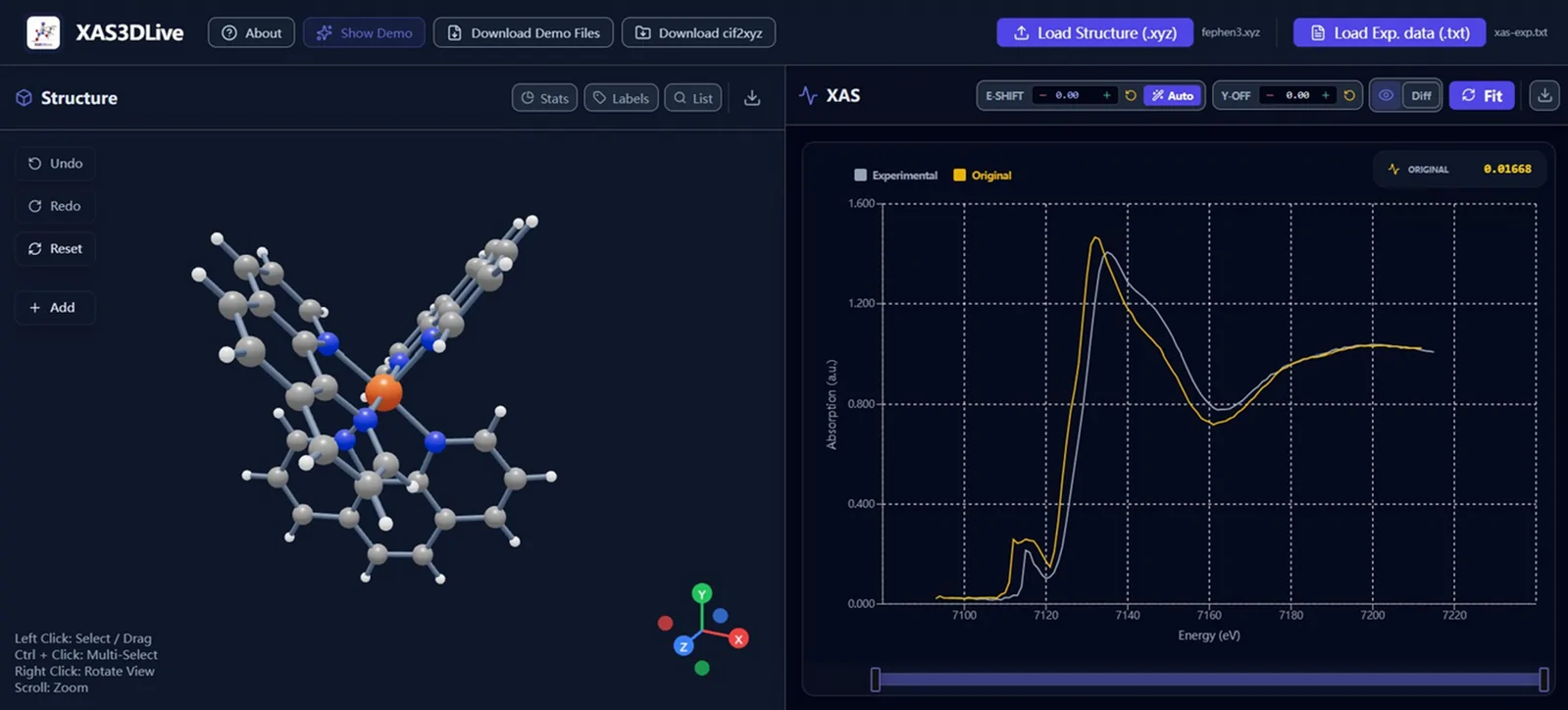
Screenshots of the *XAS3DLive* homepage displayed after selecting the ‘Show Demo’ option.

The *XAS3DLive* platform has only one interface; it has two panels, as shown in Fig. 2 ▸. The left-hand panel is the Structure panel, which provides 3D structure visualization and editing for an inputted system, while the right-hand panel is the XAS panel, which is dedicated to the display of simulated XANES or experimental data. There are several buttons around the two panels for structure editing and data analysis, which will be introduced in the following sections. Operations for the 3D structure and spectra are all taken in this interface.

### 3.2. Atomic 3D structure visualization and editing

To enable intuitive and efficient interaction with 3D structure, *XAS3DLive* implements a comprehensive suite of 3D-aware atomic selection and manipulation capabilities, including atom selection, drag-based editing, precise coordinate control in three-dimensional space, and history management.

**Figure 3:**
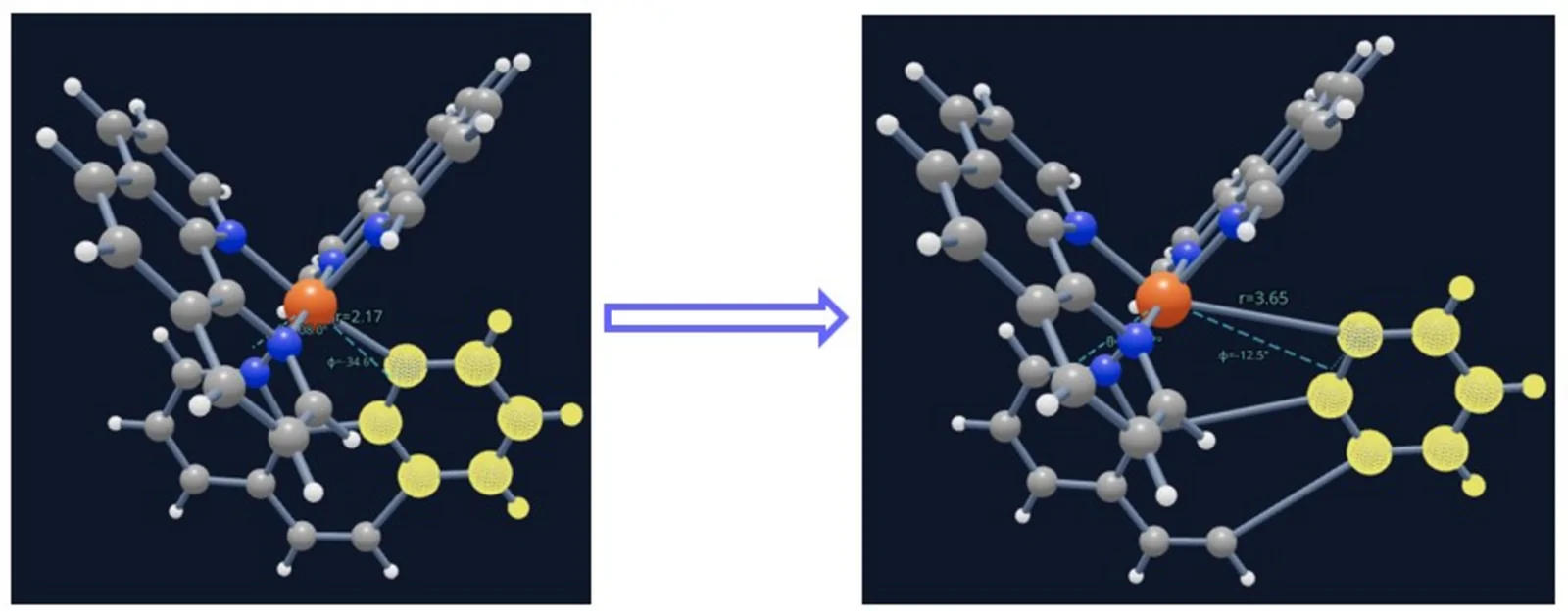
Schematic illustration of atomic cluster drag-and-drop: multiple atoms are selected to form a rigid unit.

When the structure file is imported into the platform, the Structure panel will show it instantly, and the *K*-edge XANES of the system will be displayed immediately in the XAS panel. Note that the first atom listed in the file will be taken as the absorber. The 3D structure in the Structure panel can be zoomed and rotated. Users can select one or multiple atoms in the interface; holding the Ctrl key allows atoms to be added to or removed from the current selection. Once selected, the atoms can be moved around (as shown in Fig. 3 ▸). After each modification, the corresponding XANES is immediately updated and displayed in the XAS panel. At the same time, the XANES line for the original structure of the system is still kept in the panel, so users can easily compare them and see their differences. This kind of real-time response makes it possible to observe how specific structural modification affects the spectral features. In this way, it establishes the dynamic structure–spectrum correlation of a material.

When multiple atoms are selected, the platform treats them as a ‘rigid body’, keeping their relative positions unchanged during later operations; atomic clusters or coordination units can be adjusted as a whole. This is especially helpful for structurally stable motifs, such as conjugated systems like benzene rings where bond lengths and angles tend to remain fixed. With this setup, these units can be moved or rotated together without altering their internal geometry, making it easier to examine how different configurations affect the spectra. To avoid creating unrealistic structures during interaction, the platform also adds a collision detection and warning mechanism that notifies users when a geometry may become physically unreasonable.

**Figure 4:**
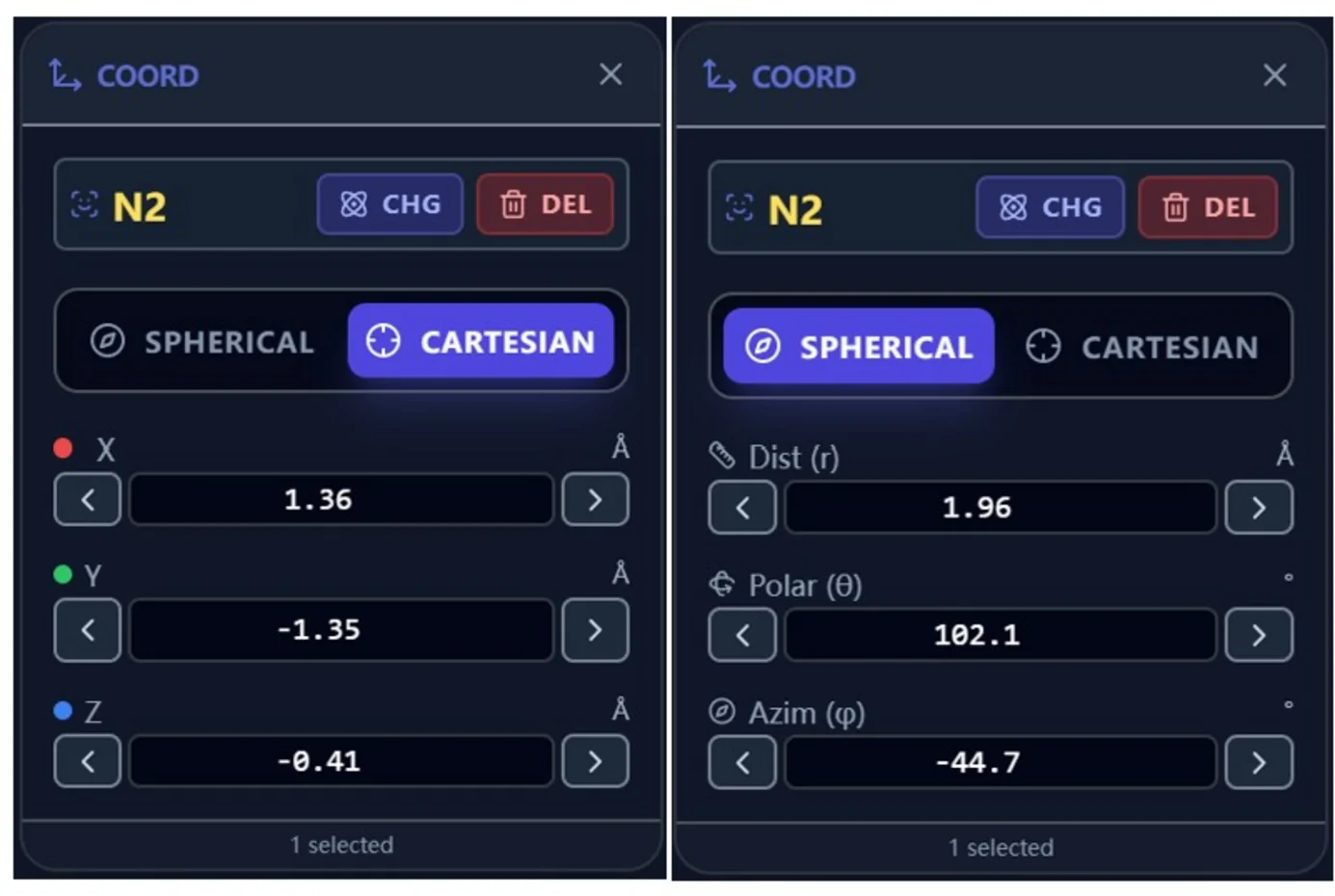
Cartesian and Spherical Coordinates panel diagram.

Besides moving atoms by dragging, *XAS3DLive* also provides precise coordinate editing functionalities. When one or more atoms are selected, a coordinate panel appears (see Fig. 4 ▸), and the atomic positions can be adjusted numerically with fine precision. The platform supports both Cartesian and spherical coordinate systems, so an atom’s position can be adjusted either by (*x*, *y*, *z*) or by radial distance *R*, polar angle θ, and azimuthal angle φ. The platform supports the real-time conversion between these two systems and bidirectional synchronization between the input values and the 3D visualization. Moreover, when the atoms move, the values in the coordinate panel will update simultaneously, keeping the visual manipulation and numerical input closely in sync and allowing responsive adjustments of the 3D structure.

To reach local structure modification and defect modeling in real material systems, *XAS3DLive* allows users to add, delete, or substitute atoms in the Structure panel. This facilitates processes such as elemental doping, formation of vacancy defects (*e.g.* oxygen vacancies), or modulation of local coordination. This atomic-level editing is closely tied to the simulation process: every structural modification instantly triggers a XANES simulation, so the updated spectra reflect instantly the local structural changes.

*XAS3DLive* incorporates an operation history management mechanism to support iterative refinement during the exploration of complex structures. All valid structural modifications are stored in a history stack, so users can easily step backward or forward through previous states using undo and redo. This not only enhances operational flexibility but also helps keep track of changes, improving the traceability of the editing process.

### 3.3. XANES analysis

*XAS3DLive* provides an XAS visualization and analysis panel, which is built on the ECharts (Li *et al.*, 2018 ▸) charting library. The panel can efficiently plot XAS curves and offers an assortment of interactive analysis tools, such as view zooming, multi-spectrum comparison, spectrum offset adjustment, and differential analysis. These tools provide a flexible and efficient environment for spectroscopic analysis.

#### 3.3.1. Multi-spectrum comparison

**Figure 5:**
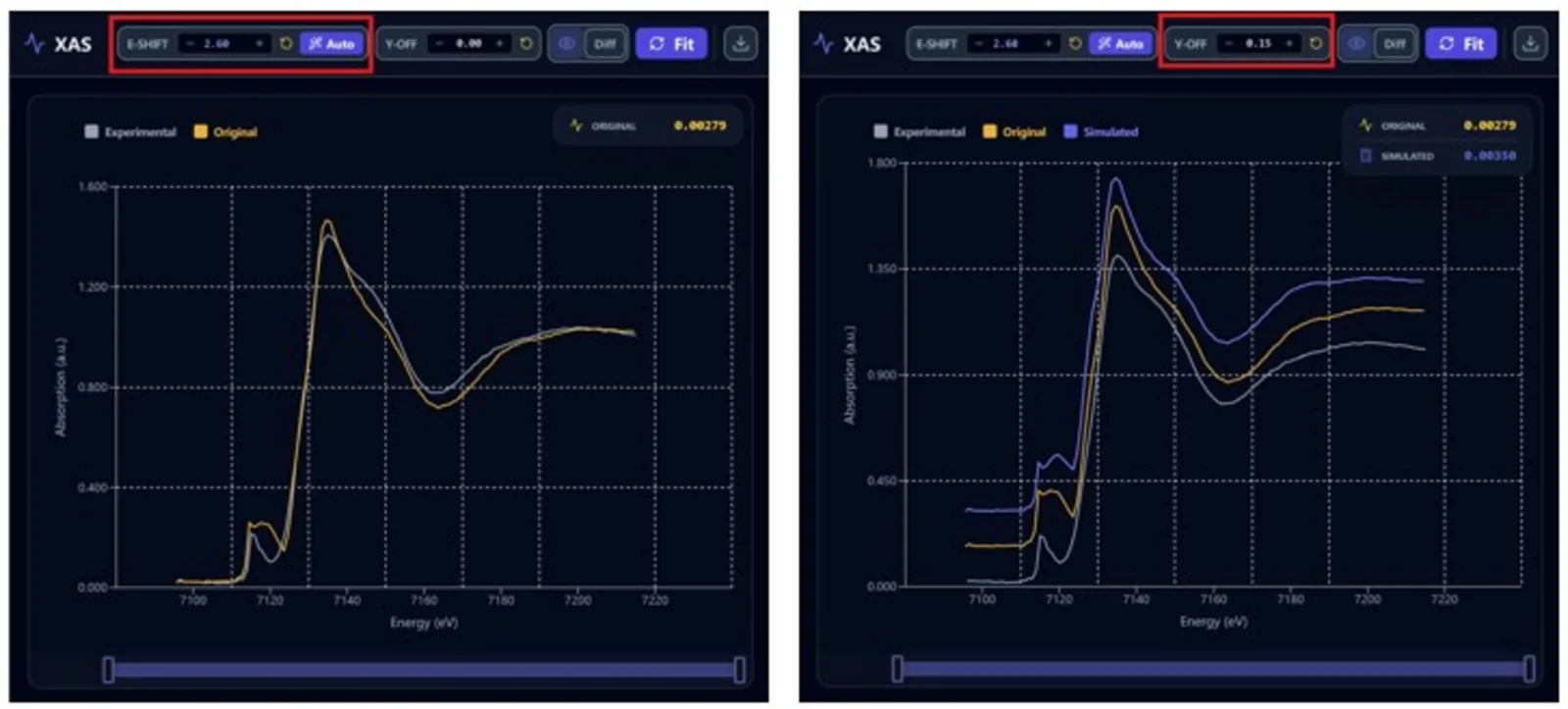
E-SHIFT and Y-Offset function diagram.

The platform allows experimental, simulated, and fitted XAS to be overlaid within the same coordinate system, making comparisons easy. It also provides tools for alignment and offset adjustment to improve comparison accuracy. The ‘Energy shift’ function allows the simulated XAS to be translated for precise alignment with the experimental XAS (as shown in Fig. 5 ▸). The ‘Offset’ function shifts spectra vertically (as shown in Fig. 5 ▸), helping to make peak positions and line shapes across different curves easier to distinguish.

#### 3.3.2. Spectral difference

**Figure 6:**
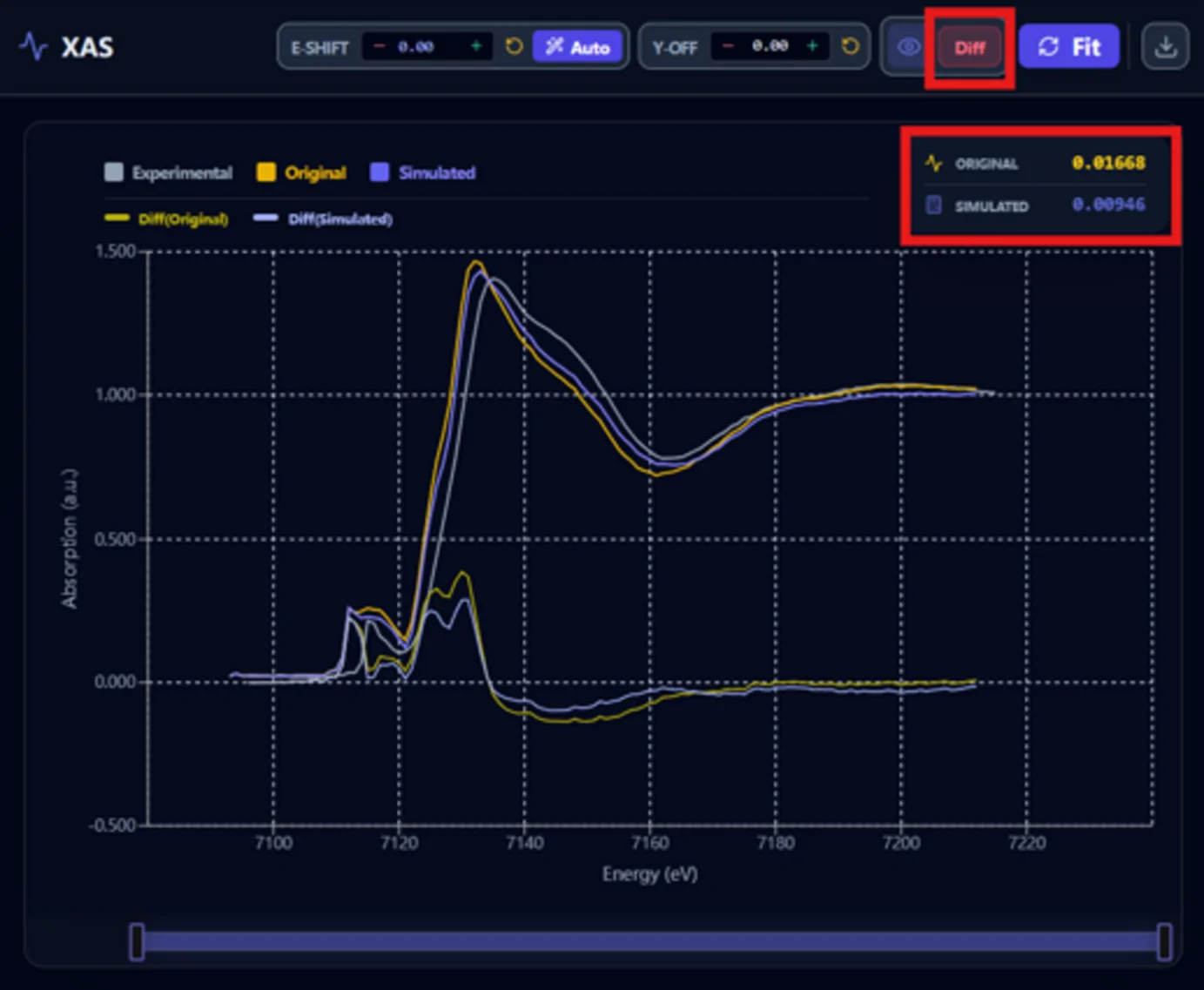
Difference spectrum and *R*-factor diagram.

The platform provides a ‘Difference spectrum’ module (as shown in Fig. 6 ▸). When the ‘Diff’ function is activated, it calculates the difference between the experimental XAS and the simulated or fitted one and displays the result in the XAS panel. This representation highlights the spectral discrepancies across the energy range, making it easier to find subtle local differences.

#### 3.3.3. Quantitative assessment

The platform adopts the *R*-factor as a metric to assess spectral agreement,
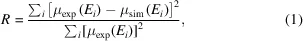
where μ_exp_(*E*_*i*_) and μ_sim_(*E*_*i*_) denote the experimental and simulated (or fitted) spectral intensities at energy point *E*_*i*_, respectively. The platform computes automatically the *R*-factor between experimental and simulated or fitted XAS and displays it in real time (as shown in Fig. 6 ▸). This value gives a global metric of how much the spectra differ; smaller values mean a better agreement—it is an intuitive and quantitative way to assess spectral consistency.

The simulated XANES given by the ML model is restricted to the range of 20 eV ahead of the absorption edge and 100 eV after it; the fitted energy range is defined as the overlap between the simulated and experimental data. To resolve the possible energy shift of experimental data in measurement and inconsistent normalization treatments to simulated and experimental data, the experimental spectra are shifted within an energy range [−5 eV, 5 eV] and normalization in the range [0.7, 1.5] during the fitting process to achieve the best convergence of two spectra.

### 3.4. *Cif2xyz* toolkit

The platform provides the *cif2xyz* toolkit, which can convert crystal structure file (CIF) format to the molecular structure format (*XYZ*). This tool can automatically parse lattice parameters and atomic position data from CIF files and convert the periodic structure into Cartesian coordinates, which is accepted in *XAS3DLive* for 3D visualization and XAS simulation.

To further improve the security and robustness of the platform, the current implementation of the *cif2xyz* converter will be removed from the web interface in future releases to eliminate potential security vulnerabilities. Instead, this functionality will be migrated to the backend, where there are more comprehensive security measures.

## 4. Application to Mn-doped Co_3_O_4_

Cobalt tetroxide (Co_3_O_4_), a typical spinel-type transition metal oxide, has been widely studied in energy storage and conversion. This is largely due to its good electrochemical properties, rich redox activity, and low cost. To further improve its catalytic performance, elemental doping is often used to tune the electronic structure and adjust active sites. Previous studies have shown that Mn incorporation into the Co_3_O_4_ lattice can produce the Jahn–Teller effect and influence the valence distribution of Co (Zhan *et al.*, 2025 ▸). At the same time, it may introduce lattice defects and create additional active centers. These combined effects can lead to a noticeable enhancement in catalytic activity. We take here Mn-doped Co_3_O_4_ as a representative system to evaluate the performance of the *XAS3DLive* platform.

**Figure 7:**
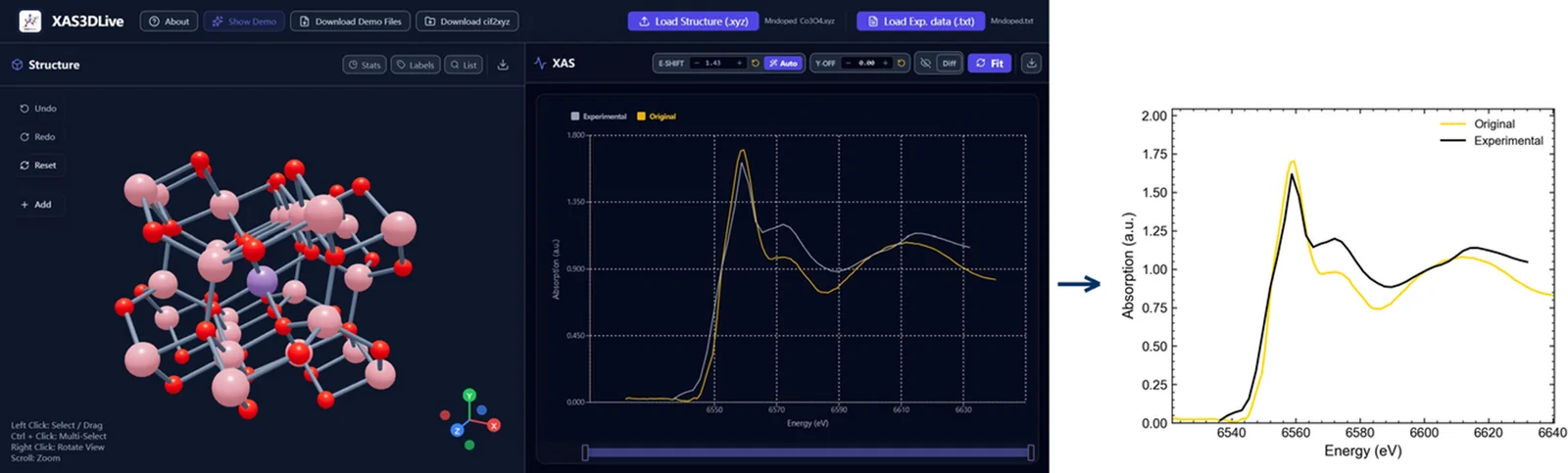
Left: screenshots of *XAS3DLive*, showing the imported Co_3_O_4_ system with the central Co atom substituted by Mn, alongside the experimental XAS of Mn-doped Co_3_O_4_ displayed in the XAS panel. Right: publication-quality XANES plot, showing the experimental spectrum together with the corresponding simulated spectra for quantitative comparison, with readable axes, energy range, and legend.

We first imported into *XAS3DLive* the Co_3_O_4_ crystal structure, taken from the XASDB library (Song *et al.*, 2025 ▸), and then replaced the central cobalt atom with a manganese atom; the simulated Mn *K*-edge XANES labeled ‘Original’ showed up immediately in the XAS panel. When the experimental data were imported, the experimental Mn-doped Co_3_O_4_ XANES, digitized using *WebPlotDigitizer* (Rohatgi, 2026 ▸) from the published figure in the work of Ke *et al.* (2020 ▸), was compared with the simulated XANES. As shown in Fig. 7 ▸, a big difference is observed between the simulated and experimental XANES, and the divergence still exists when we align the spectrum by energy shift. To find the effect of structural perturbation on spectral response, we first moved the outermost oxygen atom away from the central atom, and found that the simulated XAS exhibits only minor changes. This is expected, since the atoms in the outer shell of the absorber contribute little to the photoelectron scattering. Then, considering that Mn dopants in Co_3_O_4_ typically occupy an octahedral (six-coordinate) environment, we selected one first-nearest-neighbor oxygen atom in the equatorial plane and moved it around randomly—a significant change in the simulated XAS can be seen. In particular, when this oxygen atom moved gradually closer to the absorbing atom, the characteristic peaks in the spectrum (peak positions and intensities) moved gradually closer to the experimental XAS. This demonstrates that XAS is highly sensitive to the local coordination environment and the possible bond length change trend for the Mn-doped system. We then further adjusted the polar angle of a first-nearest-neighbor oxygen atom in the axial direction in the coordinate panel; this allowed us to rotate the atom around the central atom while keeping the bond length unchanged. When the rotation angle varied within ±20°, the simulated XAS showed only negligible changes. This suggests that, within this range of angular perturbation, the spectral features are relatively insensitive to variations in local orientation.

**Figure 8:**
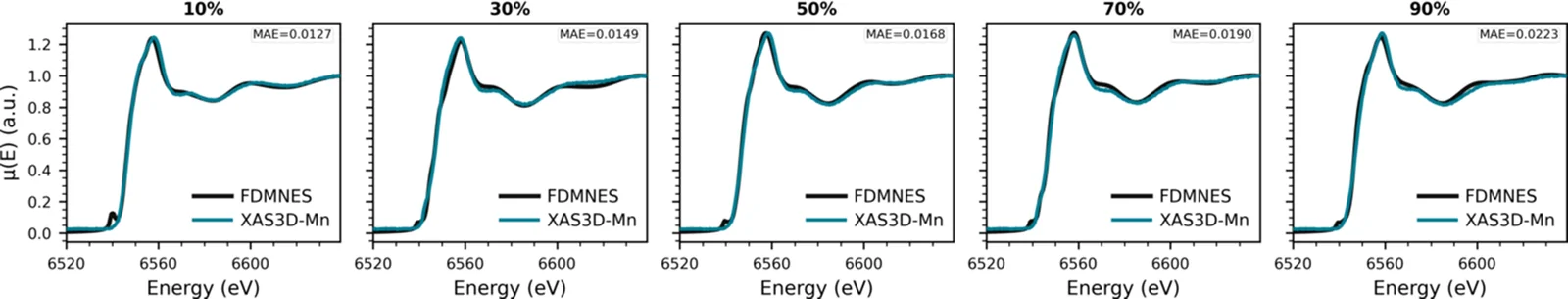
Comparison of representative Mn *K*-edge XANES spectra selected from different mean absolute error (MAE) quantiles (10%, 30%, 50%,70% and 90%) at 3256 local perturbed configurations of Mn-doped Co_3_O_4_, simulated by *XAS3DLive* and *FDMNES*.

To assess the accuracy of the XAS simulation under atomic-level manipulation, we constructed a perturbation dataset to systematically capture local structural variations. Specifically, we chose a lateral nearest-neighbor oxygen atom, an axial nearest-neighbor oxygen atom, and a nearest-neighbor cobalt atom around the absorbing manganese atom as perturbation objects, and parameterized their positions within the polar coordinate system (*R*, *Polar*, *Azim*), where *R* is the distance between the selected atom and Mn, *Polar* is the polar angle, and *Azim* is the azimuth angle. Regular grid sampling was performed around the initial configuration of these atoms; the *R* step size was set to 0.05 Å and both the *Polar* and *Azim* step sizes were set to 5°, with five steps being sampled on each side of the equilibrium position. Consequently, a total of 3256 locally perturbed structures were obtained where the unphysical ones are excluded. We performed an Mn *K*-edge XANES simulation for these structures using *FDMNES*, with the parameter settings the same as those used for training datasets; the *FDMNES*-simulated spectra were taken as the ‘ground truth’ here to check the accuracy of the XANES simulation given by the *XAS3DLive* platform. (The perturbation dataset has been made publicly available and can be freely accessed at https://doi.org/10.6084/m9.figshare.32871941.) A comprehensive statistical analysis was first performed on the distribution of mean absolute errors (MAE) for the 3256 simulated XANES generated by the model, yielding an average value of 0.0172; this is much better than that given by other GNN-based models (Huang *et al.*, 2025 ▸). We show in Fig. 8 ▸ the representative spectra corresponding to the 10%, 30%, 50%, 70% and 90% quantiles of MAE; as we can see, even in the highest-error quantile the platform-simulated XANES remains in close agreement with the *FDMNES*-simulated spectra, exhibiting strong consistency in both spectral features and peak positions. These results demonstrate that the quick response of the XANES given by *XAS3DLive* to the configuration of the system by atom dragging is very close to those that are simulated based on the framework of physical interpretation (*FDMNES* here). The proposed ML model achieves high accuracy and reliability in XAS simulations.

**Figure 9:**
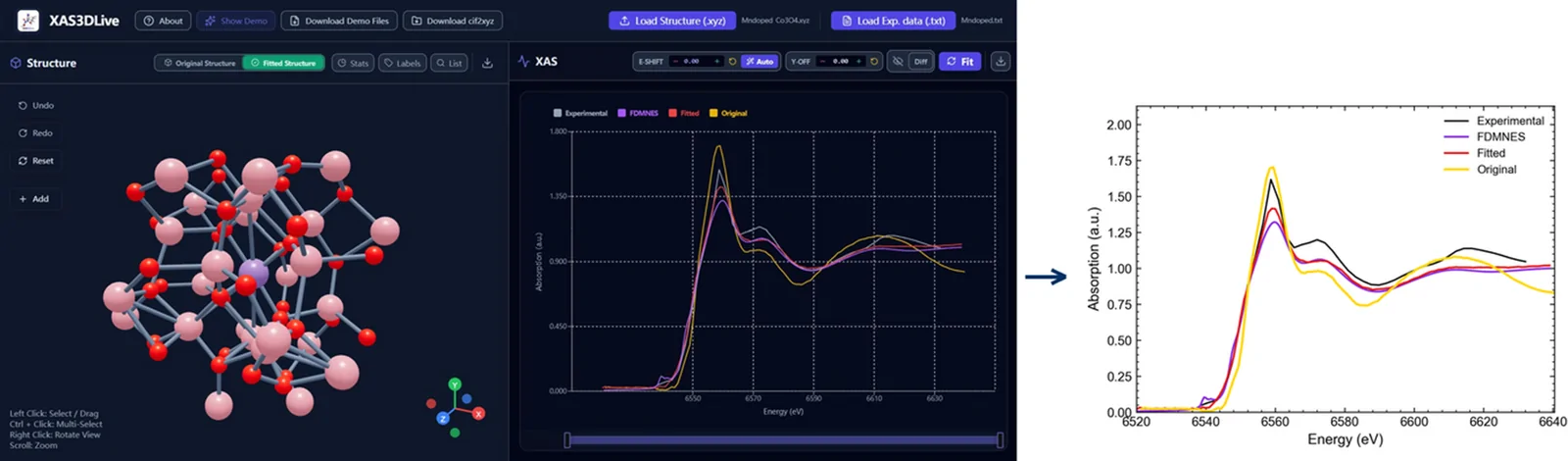
Left: screenshots of the fitted structure and Mn *K*-edge XANES for Mn-doped Co_3_O_4_ generated by *XAS3DLive*, along with the spectrum calculated by *FDMNES* using the fitted structure. Right: replot of XANES shown in the left panel.

During the structural adjustment process, users may launch a fitting operation. The fitting generally takes several or ten minutes, depending on how close the initial structure configuration is to the real one. When the fitting is done, the fitted structure of Mn-doped Co_3_O_4_ and the XANES are shown instantly in the Structure panel and the XAS panel, as shown in Fig. 9 ▸. The *R*-factor between the fitted XANES and the experimental data decreased significantly from 0.01469 to 0.00157; both the spectral profile and peak alignment show remarkable improvement compared with those of the initial structure, and the fitted XANES converges well to the experimental data. In particular, significant success is achieved for the peak located at 6590 eV, which is generally attributed to the multiple-scattering contribution from neighbor atoms. We also show in Fig. 9 ▸ the *FDMNES*-simulated XAS of the fitted geometry of Mn-doped Co_3_O_4_; very little divergence was found between the XAS given by *XAS3DLive* and that given by *FDMNES*. Quantitatively, the *R*_FDMNES_ value is 0.0006, where *R*_FDMNES_ is defined as the *R*-factor calculated between the *XAS3DLive*-simulated and *FDMNES*-calculated spectra. This small value further demonstrates the accuracy of *XAS3DLive* in the simulation of *K*-edge XANES for 3*d* transition metal complexes.

When an Mn atom is incorporated into the Co_3_O_4_ system, the central Mn atom adopts a characteristic octahedral (six-coordinate) environment, which can be resolved into two sets of inequivalent bond lengths. The fitted structure reveals that the average length of the four planar bonds shrinks to 1.89 Å, whereas the two axial bonds are elongated to 2.23 Å, yielding a pronounced difference of 0.34 Å indicative of axial distortion, which is consistent with previous reports (Zhan *et al.*, 2025 ▸). As the experimental XANES data come from the *WebPlotDigitizer* tool, the axial distortion difference may include uncertainties originating from the digitization process, but we do not think it will change the final data analysis result much. A further spin-polarized DFT+U calculation shows that the fitted structure is energetically more stable than the initial structure, with a lower total energy by 0.338 eV. This marked bond-length anisotropy reflects a symmetry breaking of the local coordination environment at the Mn site, consistent with a Jahn–Teller distortion.

## 5. Discussion

We have developed an interactive XANES simulation platform, *XAS3DLive*, devoted to providing a convenient and efficient way for users to derive the 3D structure of a material from XANES. The platform takes advantage of fast simulation from the generalized ‘structure to spectrum’ GNN model, XAS3D, and an interactive 3D atomic structure visualization/editing tool. Users can see by eye and modify the atomic structure of a material from a 3D view, instantly obtain the corresponding XANES, and check with experimental data. It is very convenient for users to find the intrinsic relationship between the local structure of the absorber and the fine structure in the spectrum by moving atoms in a material slowly and checking the behavior of the features in the spectrum. With *XAS3DLive*, users can quickly locate the most important atom(s) that affects the features in XANES, then the properties or functionality of the materials in application. This would be very useful for the design and characterization of catalysts, medicines and *in situ* reactants.

The core of *XAS3DLive* is the ‘structure to spectrum’ GNN model, XAS3D, which has very good element/structure generalizability with high XANES simulation accuracy (Zhan *et al.*, 2025 ▸; Zhan & Geng, 2026 ▸). We trained it on datasets where the structures of materials came from the Cambridge Structural Database, which is comprehensive and authoritative for small-molecule organic and metal-organic crystal structures, so the platform can be used for 3D characterization of these materials. The outreach of *XAS3DLive* for other materials has not yet been checked, but a new model trained for a specified group material is simple and acceptable; we will extend the application of the platform by training more ML models in this way. There is generally a trade-off between XANES simulation accuracy and elemental and structural generalizability of an ML model. A single model may therefore have difficulty achieving both high accuracy and broad applicability, making multiple specialized models necessary for different elemental or structural regimes. *FDMNES* was chosen for the XANES calculations in dataset construction due to its sufficient accuracy and computational feasibility; it might be replaced by other computational tools in other materials, *FEFF* for example, which works well in condensed matter. Note that, no matter which computational tool is used for XANES simulation in datasets construction, the accuracy of the simulated XANES given by an ML model trained on the datasets cannot exceed that given by these computational tools. This is a weakness but gains at thousand-times-fast-simulation speed.

Since all structures considered in this work belong to the same class of amorphous inorganic materials, a unified set of *FDMNES* parameters was adopted to generate the dataset. Such a strategy is commonly employed in XANES studies to ensure consistency among simulated spectra. Nevertheless, we know that applying an identical parameter set to all structures may introduce systematic errors, which could subsequently propagate into the ML model. To further reduce this limitation, future work will incorporate calibration strategies based on experimentally characterized standard samples and optimize the simulation parameters for different classes of materials, rather than relying on a universal parameter set. It should also be noted that the ML model is trained to reproduce *FDMNES*-generated spectra rather than experimental XAS directly. Owing to experimental uncertainties, including noise, instrumental broadening, and background artifacts, experimental XAS inevitably deviates from its theoretical counterparts. Consequently, the spectra simulated by *XAS3DLive* should be regarded as computational assistance for spectral analysis, while the final scientific conclusions should be drawn by users in conjunction with experimental conditions and data quality.

The combination of ML model and optimization algorithm makes XANES fitting simple. During fitting, the structure of a material is changed to make the simulated XANES fit best with the experimental data by minimizing the *R*-factor. It is optimal to be zero where the simulated spectrum and experimental spectrum coincide, but this is meaningless because (1) there is always an error for XANES simulated by the ML model, compared with the ground truth (here the ground truth is taken as the spectrum simulated by the non-ML-based algorithm that is used for training datasets construction, *FDMNES*), denoted as *Err*_ML_, and (2) there is always a ‘gap’ between the spectrum simulated with conventional XAS software packages and experimental data, due to the approximation and treatments made in these software—muffin-tin approximation, exchange–correlation potential correction, for example—and the mechanical disturbances and self-absorption during the measurement process, denoted as *Err*_th_. Great efforts have been taken to reduce both these errors over the decades, though they still exist. A good ML model can give a very small *Err*_ML_; it helps if the fitted structure is made more ‘stable’. The *R*-factor between ML-simulated XANES and experimental data should be at same level as *Err*_ML_ + *Err*_th_; pursuing a smaller*R*-factor is meaningless.

## 6. Conclusions

We present here a XANES simulation/fitting platform for 3D structure characterization based on a GNN-based ‘structure to spectrum’ ML model. Together with a panel for 3D structure visualization and editing, it is possible for users to modify the structure of a material and see the behavior of the spectrum instantly. A quick response of the spectrum to a new geometry of a system gives a real-time coupling between structural manipulation and spectral response, which helps users to find the intrinsic relationship between the location of special atoms and the fine structure in the spectrum. An optimization algorithm is also included in the platform for experimental data fitting. Some toolkits are also available for users to improve the manipulation experience and efficiency in XANES simulation/fitting. The capability of *XAS3DLive* is confirmed in its application to the experimental XANES analysis for the Mn-doped Co_3_O_4_ system, with observation of the Jahn–Teller effect. Moreover, the good convergence of the simulated XANES to that given by *FDMNES* for the trivial modification of atoms around the absorber confirms the generality and accuracy of the XANES simulation. Although *XAS3DLive* now supports only *K*-edge XANES analysis for inorganic or crystal systems containing 3*d* transition metals ranging from Sc to Zn, more optimized ML models trained for other edges, elements or systems can be included in the *XAS3DLive* platform to extend to more applications. *XAS3DLive* facilitates and improves the experience of XANES analysis—we expect it will help in accelerating materials exploration.
